# Family Peace

**Published:** 1856-05

**Authors:** 


### FAMILY PEACE.

1. Remember that our will is likely to be crossed every day, so prepare for it.

2. Everybody in the house has an evil nature as well as ourselves, and therefore we are not to expect too much.

3. To learn the different temper of each individual.

4. To look upon each member of the family as one for whom Christ died.

5. When any good happens to any one, to rejoice at it.

6. When inclined to give an angry answer, to lift up the heart in prayer.

7. If from sickness, pain or infirmity, we feel irritable, to keep a very strict watch over ourselves.

8. To observe when others are so suffering, and drop a word of kindness and sympathy suited to them.

9. To wait for little opportunities of pleasing, and to put little annoyances out of the way.

10. To take a cheerful view of everything, of the weather, and encourage hope.

11. To speak kindly to the servants, to praise them for little things when you can.

12. In all little pleasures which may occur, to put self last.

13. To try for "the soft answer which turneth away wrath."

14. When we have been pained by an unkind word or deed, to ask ourselves, "Have I not often done the same and been forgiven?"

15. In conversation not to exalt ourselves, but to bring others forward.

16. To be very gentle with the young ones, and treat them with respect.

17. Never to judge one another, but to attribute a good motive when you can.—*Churchman's Monthly Penny Magazine.*

